# Covid‐19 and Namaste

**DOI:** 10.1111/irv.12746

**Published:** 2020-04-21

**Authors:** Prashanth Kulkarni, Shruthi Kodad, Manjappa Mahadevappa

**Affiliations:** ^1^ Department of Cardiology Care Hospitals Hyderabad India; ^2^ Department of Haematology Saskatoon Cancer Centre Saskatoon SK Canada; ^3^ Department of Cardiology Jagadguru Sri Shivarathreeshwara University Mysuru India

**Keywords:** Covid‐19, Namaste, pandemic

To the Editor‐in‐Chief

The novel coronavirus (SARSCoV‐2) has emerged as a major pandemic stretching the healthcare resources of most countries of the world. In this context, it is imperative that social distancing and good hand hygiene is practised to stem the transmission of this highly contagious virus.

The WHO’s standard recommendations to prevent infection spread include regular hand washing, covering mouth and nose when coughing and sneezing, thoroughly cooking meat and eggs and to avoid close contact with anyone showing symptoms of this respiratory illness.^1^ Also shaking hands or any form of hand‐to‐hand contact should be avoided as cross‐transmission of organisms occurs through contaminated hands.^2^

In most countries of the World, handshake, fist bump, high five and hugs are some of the different methods of greeting each other, which leads to physical proximity and contact, facilitating rapid propagation of infections such as Covid‐19.

Alternatively, other non‐physical greeting forms can be explored like Namaste, which is used in Indian subcontinent since hundreds of years to greet people with folded hands, while maintaining a fair distance from each other [Figure 1]. An individual in addition to saying “Namaste” presses his hands together in front of the chest and respectfully greets the other person. This form of greeting does not involve any physical touch between individuals and gives a sense of parity to all the parties.^3^

**Figure 1:**
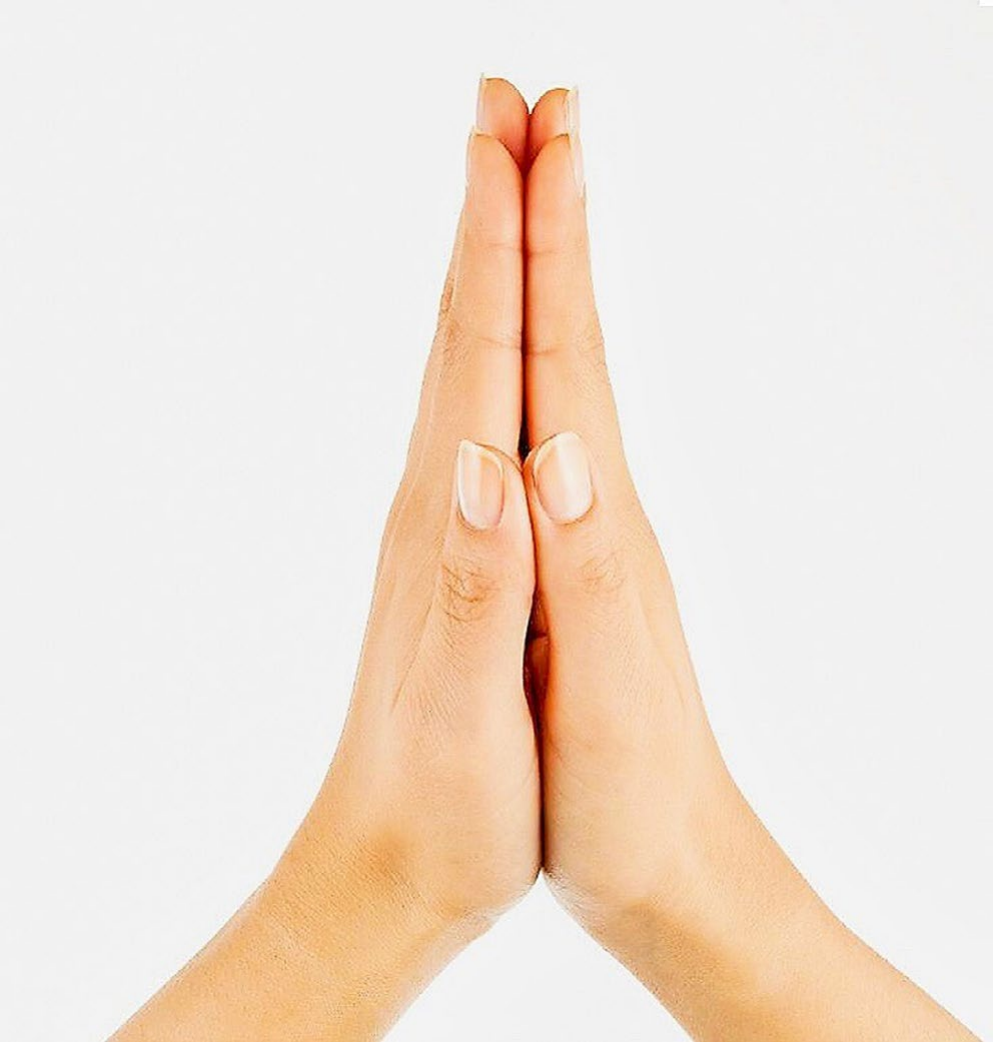
Namaste

In addition to following general principles of meticulous hand washing, rapid transmission of infections both in hospitals and the community can be overcome by adopting the no‐touch salutation Namaste and other such forms like bowing the head as done in some Asian countries.

## CONFLICT OF INTEREST

The authors declare no conflict of interest.

## AUTHORS CONTRIBUTION

Prashanth Kulkarni: Conceptualization‐Lead, Methodology‐Lead, Resources‐Lead, Software‐Lead, Writing‐original draft‐Lead, Writing‐review & editing‐Equal; Shruthi Kodad: Supervision‐Equal, Validation‐Equal, Visualization‐Equal; Manjappa Mahadevappa: Visualization‐Equal, Writing‐review & editing‐Equal.
